# Family conflict and lower morning cortisol in adolescents and adults: modulation of puberty

**DOI:** 10.1038/srep22531

**Published:** 2016-03-01

**Authors:** Jihui Zhang, Siu-Ping Lam, Alice PS Kong, Ronald CW Ma, Shirley Xin Li, Joey WY Chan, Mandy WM Yu, Junying Zhou, Michael HM Chan, Chung-Shun Ho, Albert M Li, Xiangdong Tang, Yun-Kwok Wing

**Affiliations:** 1 Department of Psychiatry, Faculty of Medicine, The Chinese University of Hong Kong, Hong Kong SAR, China; 2 Shenzhen Research Institute, The Chinese University of Hong Kong, Hong Kong SAR, China; 3 Department of Medicine and Therapeutics, Faculty of Medicine, The Chinese University of Hong Kong, Hong Kong SAR, China; 4 Department of Psychology, The University of Hong Kong, Pokfulam Road, Hong Kong SAR, China; 5 Department of Chemical Pathology, Faculty of Medicine, The Chinese University of Hong Kong, Hong Kong SAR, China; 6 Department of Paediatrics, Faculty of Medicine, The Chinese University of Hong Kong, Hong Kong SAR, China; 7 Sleep Medicine Center, Mental Health Center, West China Hospital, Sichuan University, Chengdu, China

## Abstract

We aimed to explore the association between family conflict and HPA axis activity, especially with respect to the potential modulating effect of puberty. A total of 205 adolescents and 244 adult parents were recruited. Family conflict was assessed by the family conflict subscale of the Family Environmental Scale and serial salivary cortisol was measured in all participants. A marginally lower AUC_g_ at 30 minutes after wake up in the morning and a significant lower AUC_g_ at 60 minutes and 90 minutes in adult parents with high family conflict was found when compared to those with low family conflict. In adolescents, there were significant interaction effects between pubertal status and family conflict on AUC_g_ (interaction p values <0.05). Among the adolescents with low family conflict, those at late/post pubertal status had higher AUC_g_ than their pre/early pubertal counterparts but this difference was not observed in the adolescents with high family conflict. Adverse family environment is associated with HPA axis dysfunction in adults and late/post pubertal adolescents and pubertal maturation plays a critical role in modulating the association between family environment and HPA axis function.

Family conflict is a chronic stressor in daily life and a common risk factor for poor health in both parents and their children^1^. Although conventional impression opined that parent-child conflict, a common type of family conflict, is more common during adolescence than during childhood, a meta-analytic review compared whether the source of data reported by parents or by children will influence levels of conflict across adolescence. It has concluded that puberty is associated with a decline in the rate of conflict as reported by both parents and adolescents. However, only adolescents but not parent report a significant increase in the conflict affect (i.e. emotional intensity of disagreement)^2^, which suggests that adolescents tend to perceive a high level of emotional intensity of parent-child conflict during adolescence even though the frequency of the conflict declines with pubertal progression. These findings imply that family conflict is modulated across adolescence period with increased emotional intensity amplified by the pubertal maturation.

Cortisol level is an indicator of the function of hypothalamic–pituitary–adrenal (HPA) axis with a significant circadian variability^3^. Dysfunction of daytime cortisol activity is associated with a series of stress-related disorders, including insomnia^4^, depression^5^,^6^, anxiety disorders^7^, post-traumatic stress disorder (PTSD)^8^, and obesity^9^. A few studies have shown that the association of chronic stress with diurnal salivary cortisol secretion, however, varies from no effect to a blunted or enhanced response^10^,^11^,^12^. These inconsistent findings may be partially explained by the variations of the measurements, type of the stressors studied, and samples in different studies^13^. Family conflict, as a common chronic stressor in daily life, has been found to be associated with the cortisol levels across child, adolescent and adult populations^14^,^15^,^16^,^17^,^18^,^19^, which might be the potential pathway underlying the detrimental effects of family conflict on mental and physical health. In general, individuals with high family conflict seem to have a lower cortisol level upon awakening and a flatter diurnal cortisol slope, albeit there remains a discrepancy in the existing findings^14^,^15^,^16^,^17^,^18^,^19^. Some limitations in the previous studies should be noted. First, the association between family conflict and cortisol level should be replicated in different developmental periods, given that young children and adolescents undergo rapid and dramatic changes in their physical and psychosocial development. For example, it has been suggested that puberty significantly modulates the association between adversity and cortisol level^17^,^20^,^21^. Second, it has been shown that there are significant ethnic differences in the diurnal cortisol levels^18^. In particular, there is a lack of studies investigating the effects of adverse family environments on HPA axis across different ethnic groups. Third, most of the previous studies used only 1–2 time points of cortisol levels or the cortisol level before and after a challenge test. The cortisol awakening response (CAR) represents the natural response of HPA axis to daily awakening, which might have important implications in the pathophysiology of various mental and sleep disorders^19^. In addition, previous studies have suggested that females have a higher cortisol response to acute stress and awakening on workdays (indicating work-related stress) than males^22^,^23^,^24^ but little is known whether there are sex differences in HPA axis response to family conflict.

In this study, we hypothesized that family conflict is associated with lower diurnal cortisol profiles in the Chinese population. In view of the potential roles of puberty in modulating the effects of adverse environments on HPA axis activity, we hypothesized that pubertal status may modulate the association between family conflict and diurnal cortisol profiles. As previous studies have suggested a potential role of puberty in modulating the parent-child conflict^2^, we also aimed to explore whether there is an interaction effect between the level of family conflict and pubertal status on the diurnal cortisol profiles. In addition, we included the parents in this study in order to examine the effect of family conflict on HPA axis activity in adults. Finally, we also explored whether there are sex differences in HPA axis response to family conflict.

## Methods

The current study was part of a case-control family study of insomnia and mental disorders^4^,^25^. Adolescent probands were selected from the school-based survey study (2008–2010) based on the presence/absence of sleep problems using a risk-stratification approach^25^, Their biological parents and full siblings over 6 years old were also invited to take part in this study. As the study originally aimed to recruit as many adolescents with insomnia as possible, the rate of insomnia in the adolescent group was higher than those figures reported in the epidemiologic studies. In the original sample, a total of 236 probands and 562 first degree relatives were recruited for the clinical assessments, with a response rate of 82.8% 25. Due to the limited resources, a total of 212 children and adolescents (probands and siblings) and 244 middle-aged adults (parents) were randomly selected to complete salivary cortisol assessments, representing 56.1% and 58.1% of adolescents and adults respectively who were recruited into this phase 2 family study. Seven out of the 212 adolescents with missing data on their pubertal status were excluded from the analyses^4^. A total of 205 adolescents were included into the final analyses. For further details about the sample recruitment, please refer to our previous publications^4^.

## Ethical Statement

The protocols of this study were approved by the Institutional Ethics Review Committee of the Joint Chinese University of Hong Kong – New Territories East Cluster Clinical Research Ethics Committee (The Joint CUHK-NTEC CREC). The current study was conducted in accordance with the approved protocols and the Declaration of Helsinki. Participants aged 18 years old or above gave written informed consent. For the participants aged under 18 years old, their parent(s)/caregiver(s) gave written consent and the participants gave written assent to take part in the study.

## Measures

### Assessment of family conflict

The Family Environment Scale (FES) is a scale that is designed to measure the social-environmental characteristics of a family, including cohesion, expressiveness, family conflict, independence, achievement orientation, intellectual-cultural orientation, moral religious emphasis, organization, and control. The family conflict subscale in the Family Environment Scale (FES) consists of 9 items to assess the amount of openly expressed anger and conflict among family members in daily life, such as fighting, being openly angry, and losing tempers. The Chinese version of FES (FES-CV) has been shown to have good validity and reliability^26^. The FES-CV was completed by parents. The total score of family conflict subscale of FES ranged from 0 to 9, with a mean ± standard deviation (SD) = 4.06 ± 2.27 in the current sample. Those families with a score on the FES-CV family conflict subscale higher than 1 SD (cut-off =6) were considered as high conflict families, while the rest were considered as low conflict families.

### Ascertainment of mental disorders and insomnia disorder

Structured Clinical Interview for DSM-IV Axis I Psychiatric Disorder (SCID-I) was employed to assess both parents and sibling(s) aged 18 or above. Diagnostic Interview Schedule for Children-Version 4 (DISC-IV) was employed to assess adolescents (probands) and their sibling(s) below 18 years of age^27^. The diagnosis of insomnia disorder was determined by the clinicians on the basis of Diagnostic and Statistical Manual of Mental Disorders, the fourth edition (DSM-IV) criteria for insomnia disorder.

### Pubertal status

Pubertal status was assessed by the Tanner pubertal self-assessment questionnaire for children and adolescents. This validated self-report scale has an excellent agreement with the rater-rated assessment in Chinese adolescents^28^. Participants with Tanner stages of 1–3 (mean age (SD) = 12.6 (1.9) years for males and 11.9 (2.3) years for females) were considered as having the pre/early pubertal stages, while those participants with Tanner stages of 4–5 (mean age (SD) = 15.9 (2.6) years for males and 14.9 (2.5) years for females) were considered as having the late/post pubertal status. As there were only 16 cases with Tanner stage of 1, they were not divided into a separate group.

### Serial salivary cortisol and data cleansing

Participants were instructed to collect their salivary samples using Sarstedt Salivettes^®^ at the following time points: immediately after awakening (0 min, T1), and 30 minutes (T2), 60 minutes (T3) and 90 minutes (T4) after awakening, noon time (T5), 4:00 pm (T6) and 10:00 pm (T7) during a free day (weekends or holidays) while they were wearing the actigraphy for the concurrent measurement of their sleep pattern and sleep quality^4^. Eating, drinking, and teeth brushing were not allowed 30 minutes before the sample collection. Participants were asked to record the sampling time and their wake up time in actigraphy and/or sleep diary. Those samples of the first 4 time points (T1–T4) collected outside a margin of 5 minutes before or after the time specified in the protocol were considered as missing. Salivary samples were stored at −80 °C. Salivary cortisol concentrations were measured on an automated electro-chemiluminescence immunoassay system. The laboratory is accredited by the Australian National Association of Testing Authorities. The within-batch and between-batch precision coefficients of variation (n = 10) were <9% and <18%, respectively. Cortisol values beyond 3 SDs were considered as outliers and hence were coded as missing data. The missing data in those participants with at least two valid cortisol values were estimated by the expectation maximization method in Lisrel 8.7 for Windows (Lincolnwood, IL) with the adjustment of age and sex^4^. A total of 128 (10.4%) and 94 (7.6%) salivary samples were coded as missing in adolescents and adults, respectively. The salivary cortisol values of missing samples were estimated by imputation as described above.

### Estimation of Cortisol Awakening Response (CAR)

Two formulae proposed by Pruessner *et al.*^29^ were employed to calculate the area under the curve (AUC) of cortisol awakening response (CAR) including AUC with respect to ground (AUC_g_) and AUC with respect to increase (AUC_i_). The AUC_g_ was considered as an estimate of the total cortisol secretion (overall intensity) throughout the day while AUC_i_ was suggested to be a measure of dynamic change of the cortisol awakening response. The AUC_i_ and AUC_g_ were calculated using the salivary cortisol values at the first 4 time-points (i.e. immediately after awakening, and 30 minutes, 60 minutes, and 90 minutes after awakening).

## Statistics

**Figure 1:**
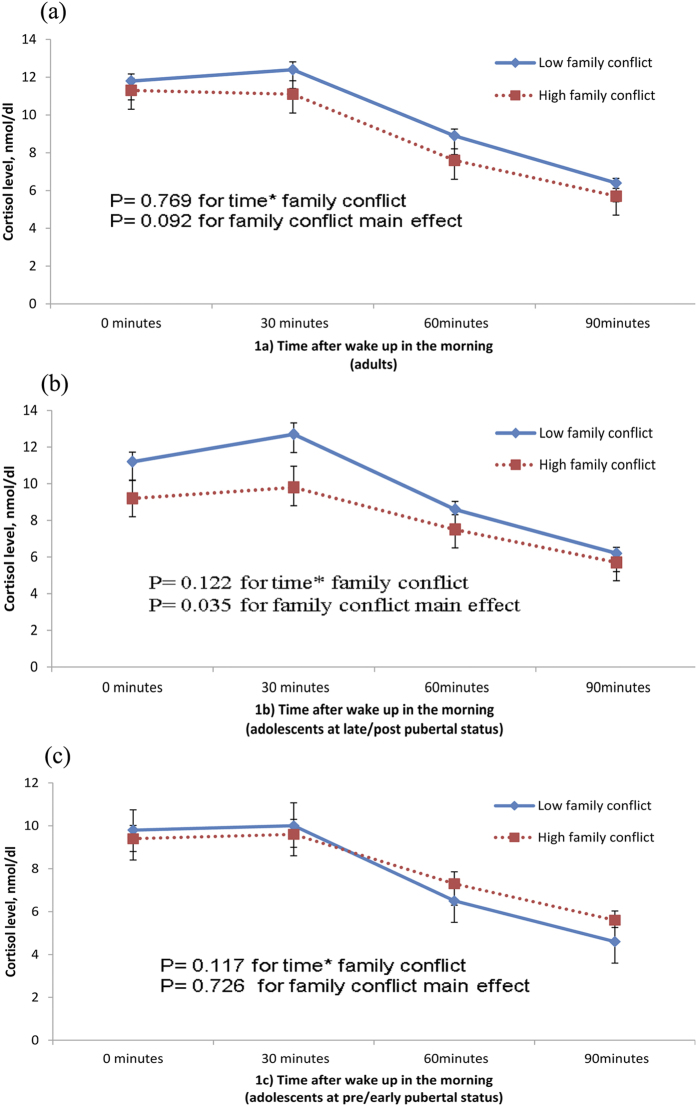
Salivary Cortisol levels within 90 minutes after awakening in different groups. The solid lines indicate cortisol levels within 90 minutes after awakening for participants from low family conflict groups. Group differences were tested by repeated measures ANOVA. (**a**) shows that adult parents with high family conflict had marginally lower cortisol levels than adult parents with low family conflict. (**b**) shows that cortisol levels were lower in adolescents at late/post puberty with high family conflict than those adolescents at late/post puberty with lower family conflict, which were generally consistent with findings from adults. However, there were no differences in cortisol levels between high family conflict and low family conflict in adolescents at pre/early puberty (**c**). The findings by using mixed effect modeling (XTREG module of Stata version 14.0) were consistent with the findings by using repeated measures ANOVA, which show that the effect of family conflict on cortisol levels within 90 minutes upon awakening (4 time points) was −1.51 ± 0.69 for adults (z value = −2.17, p = 0.03), −1.95 ± 0.89 for adolescent at late/post puberty (z value = −2.17, p = 0.03), and 1.03 ± 0.93 for adolescent at pre/early puberty (z value = 1.10, p = 0.27).

Descriptive statistics were presented as percentages for discrete variables and as means (standard deviations) for continuous variables. The participants were divided into two groups first, adults and adolescents. The comparison between participants with high and low family conflict with respect to socio-demographic features, pubertal status and clinical characteristics was performed by independent sample *t*-test and chi-square test, wherever appropriate. Morning awakening cortisol curves of the participants with high and low family conflict in different subgroups were examined by repeated measures ANOVA (Fig. 1), which allowed us to compare the differences in the repeated measures of cortisol level within 90 minutes after awakening. In view of the potential correlations among participants within a family, Generalized Estimating Equation (GEE) was employed in all analyses except for Fig. 1 (as GEE model cannot be applied to the repeated measure data) to compare the differences in the diurnal salivary cortisol profiles between the participants with low and high family conflict^30^. GEE model is able to take into account the random effect derived by multiple individuals from the same family. The effects of a series of potential confounding factors, including age, sex, current insomnia disorder, current depressive disorders, current anxiety disorders, chronic medication use, chronic medical condition, seasonality, and rise time in the morning when collecting saliva sample, were also adjusted in the GEE models. Our previous study has shown that pubertal status was a significant moderator of HPA axis response to insomnia^4^. Therefore, the interactions between pubertal status (pre/early pubertal status vs. late/post pubertal status) and family conflict (low family conflict vs. high family conflict) on the diurnal salivary cortisol profiles were also examined in the GEE model. In view of the potential sex differences in HPA axis reactivity associated with the level of family conflict, interaction terms with sex and family conflict were additionally tested in repeated measures ANOVA and GEE model. In view of the concern that using the expectation on maximization method to estimate missing values of cortisol level may have led to potential bias in the CAR analysis and a higher rate of insomnia, XTREG and GLM modules of Stata version 14.0 (Stata Corporation, USA) were further employed to confirm the findings (additional analyses, Supplementary Tables 1–3). A p value < 0.05 was considered statistical significance.

## Results

### Sample characteristics between participants with low and high family conflict

High conflict families (n = 47) had a lower score on the cohesion subscale (6.47 ± 2.21 vs. 7.92 ± 1.00, p < 0.001) and organization subscale (6.54 ± 1.68 vs. 5.71 ± 1.94, p = 0.009) but higher scores on the independence (6.34 ± 2.41 vs. 5.52 ± 1.24), achievement orientation (6.32 ± 2.62 vs. 5.20 ± 1.73, p = 0.009), and control (4.57 ± 2.17 vs. 3.87 ± 2.0, p = 0.046) subscales, when compared with low conflict families (n = 120). No differences in intellectual cultural orientation, active-recreational orientation, and moral religious emphasis subscales.Table 1 shows the characteristics of the participants with high and low family conflict. The adolescents with high family conflict had very similar demographic, clinical and other characteristics to those with low family conflict, except for the season of sample collection. Recruitment and salivary sample collection were more likely to be completed during winter and spring and less likely to be completed during summer for adolescent participants with high family conflict. No association was found between the level of family conflict and pubertal status (p = 0.24).

Adults with high family conflict had comparable demographic and other clinical characteristics to those with low family conflict except that those with high family conflict had a higher rate of current mood and anxiety disorders as compared to their counterparts (13.1% vs. 3.8% for current mood disorders and 9.8% vs. 2.2% for current anxiety disorders, p < 0.05).

### Family conflict and diurnal cortisol levels

Table 2 shows the differences in the diurnal cortisol levels between adults with low and those with high family conflict after adjusting for potential confounding factors. In general, adults with high family conflict had lower morning cortisol levels than their counterparts at 30 minutes (mean difference ± SE = −1.6 ± 0.9 nmol/l, p = 0.09) and 60 minutes (mean difference ± SE = −1.5 ± 0.6 nmol/l, p = 0.02) after awakening. The ACU_g_ at 60 minutes (mean difference ± SE = −2.5 ± 1.3 nmol/l, p = 0.04) and at 90 minutes (mean difference ± SE = −3.7 ± 1.8 nmol/l, p = 0.03) were significantly lower in adults with high family conflict than those with low family conflict. There were no differences in AUC_i_ at 30 minutes between 2 groups.

In the preliminary analysis, there were no differences in the salivary cortisol variables between adolescent participants with high and low family conflict. Previous studies have shown that HPA axis may respond to the adversity differently across the pubertal stages^17^,^20^,^21^. We speculated that puberty might modulate the association between family conflict and morning cortisol level. We therefore further tested whether there were significant interactions between pubertal status and the level of family conflict. Table 3 shows that there were significant interaction effects between pubertal status and the level of family conflict on the cortisol levels at 30 minutes (p = 0.05), 60 minutes (p = 0.015), and 90 minutes (p = 0.032), and AUC_g_ at 90 minutes (p = 0.006). These findings suggested that pubertal status moderated the effects of family conflict on the cortisol secretion upon awakening. In pre/early pubertal adolescents, similar patterns of diurnal cortisol profiles were found between those with high and low family conflict. However, in late/post pubertal adolescents, those with high family conflict had a lower level of cortisol in the morning when compared with their counterparts. On the other hand, among adolescents with low family conflict, those in the late/post puberty group had a significantly higher AUC_g_ than those in the pre/early puberty group. However, in adolescents with high family conflict, pubertal status was not found to be associated with AUC_g_ and cortisol levels. Further analyses based on the GEE model revealed that there were no significant interactions (p > 0.20) between sex and the level of family conflict on all the cortisol parameters in both adults and adolescents.

Results shown in Fig. 1 were generally consistent with the findings in Table 2 and 3. There was a main effect of the level of family conflict on the morning awakening cortisol secretion. Morning awakening cortisol level was nearly significantly lower (p = 0.092) in the adults with high family conflict. In addition, late/post pubertal adolescents with high family conflict had significantly lower morning cortisol levels as compared to their counterparts (p = 0.035). However, this main effect was not observed in the adolescents at the pre/early pubertal status (p = 0.73). There were no significant interactions between time and family conflict in the cortisol morning awakening response in all age groups (p > 0.05). Further analyses using repeated measure ANOVA revealed that there were no significant interactions (p > 0.20) between sex and family conflict on all the cortisol parameters in adults and adolescents.

### Additional analyses

To address the concern that the imputation for missing value of cortisol might have led to bias in the CAR analysis, we ran additional analyses using the XTREG module of Stata version 14.0 (Stata Corporation, USA), with each time point of cortisol nested within each participant. This method allows those participants with missing data to be included in the analyses. All multilevel models were estimated on the basis of all the available data. The mixed effect modeling showed that the effect of family conflict on the cortisol levels within 90 minutes upon awakening (4 time points) was −1.51 ± 0.69 for adults (z value = −2.17, p = 0.03), −1.95 ± 0.89 for late/post pubertal adolescents (z value = −2.17, p = 0.03), and 1.03 ± 0.93 for pre/early pubertal adolescents (z value = 1.10, p = 0.27). There results were consistent with the findings by estimating missing values with the expectation maximization method.

In addition, to address the potential recruitment bias due to the original study design, we weighted the prevalence of insomnia in this study based on the local epidemiologic data from our previous studies (10% in adults and 6% in adolescents)^31^,^32^ The results are presented in the supplementary Tables 1–3. In adults, family conflict level was found to be negatively associated with the awakening salivary cortisol level at 30 minutes and 60 minutes, and AUC_g_ at 30 minutes, 60 minutes, and 90 minutes. In late/post pubertal adolescents, family conflict level was found to be negatively associated with the awakening salivary cortisol level at 30 minutes and at noon, and AUCg at 30 minutes and 90 minutes. However, no association was found between the level of family conflict and diurnal cortisol level in pre/early pubertal adolescents. These findings based on the weighted prevalence of insomnia are consistent with the results shown in Tables 2 and 3.

## Discussion

We found that participants with high family conflict had a lower level of cortisol secretion in the first 1.5 hours after awakening, which was independent of the concomitant physical, sleep and mental disorders. In particular, this phenomenon was only observed in the late pubertal adolescents and middle-aged adults (parents) but not among pre/early pubertal adolescents. Furthermore, the cortisol levels in the morning (within 90 minutes after waking up) in adolescents increased with the pubertal maturation in adolescents with low family conflict but not among those with high family conflict. In both adults and adolescents, males and females respond similarly to family conflict in terms of the morning cortisol level. As the sensitization of HPA axis during the pubertal maturation might modulate stress reactivity and brain maturation in adolescents^33^, the dysregulated HPA activity in those late/post-pubertal adolescents with high family conflict might have far-reaching negative impacts on their mental and physical health development^14^,^33^.

By collecting serial daytime salivary samples, we demonstrated that high family conflict was associated with a lower overall level of cortisol secretion (AUC_g_) in the morning but not with the dynamic changes of morning cortisol level (AUC_i_) and daytime cortisol levels. The findings on the lower morning cortisol levels after awakening (AUC_g_) in the individuals with high family conflict were consistent with that of most of the other studies^14^,^15^,^16^,^17^,^18^,^19^. Although previous studies have shown that these individuals had a higher cortisol level in the latter part of the day, our study did not replicate this finding^14^,^34^. The exact mechanism underlying the insignificant association between the level of family conflict and cortisol level in the latter part of the day in our study is unclear. We speculated that daytime cortisol may be less reactive than morning cortisol in response to the environmental adversities. In addition, it has been shown that AUC_g_ post awakening is more stable across different days than daytime cortisol levels, which indicates less measurement errors in AUC_g_^35^.

There are several possible explanations for the association of high family conflict with lower morning cortisol in adults and late/post pubertal adolescents. First, puberty may modulate the effect of family conflict or other stressors on HPA axis activity. A number of studies have investigated the modulation of pubertal development on HPA axis response to stress tests, such as public speaking^20^, Trier Social Stress Test^21^, psychosocial stress^36^, and general adversities^17^. For example, Bosch *et al.* have suggested that adversities during ages of 6–11 years are associated with higher cortisol levels while adversities during ages of 12–15 years are associated with lower cortisol levels upon the challenge of Groninger Social Stress Test^17^. In this regard, our findings are consistent with the previous observations that pubertal development is a key modulator of HPA axis response to stress.

The second explanation is that the normal pubertal increase of cortisol levels in the morning was suppressed in those adolescents with high family conflict. Recent normative data from the Healthy Lifestyle in Europe by Nutrition in Adolescence (HELENA) study have shown that basal cortisol level increases across puberty (from 10.7 ug/dl at age 13 years to 14.4 ug/dl at age 16 years for boys, and from 11.1 ug/dl at age 13 years to 15.9 ug/dl at age 16 years for girls)^37^. In other words, normal pubertal progression is associated with a significant surge in the basal cortisol level. Similarly, we found that among adolescents with low family conflict, those at late/post pubertal status had significantly higher cortisol levels in the morning and AUC_g_ than those at the pre/early pubertal status. On the other hand, significant associations between high family conflict and lower AUC_g_ were only found in the late/post pubertal adolescents but not those in the pre/early puberty group (Fig. 1). These findings suggested that high family conflict might suppress the normal surge of post awakening cortisol levels (AUC_g_) at late puberty. Laboratory studies have shown that cortisol response to post-defeat model increases from early puberty to mid puberty in healthy male golden hamsters. However, this kind of increase is significantly stunted in subjugated hamsters^38^.

The third explanation for the differences in the HPA responses associated with early and late puberty is possibly the differential exposure to the family conflict between young and older adolescents. However, our data showed that pre/early pubertal adolescents did not differ from their late/post pubertal counterparts in terms of the rate of high family conflicts. Previous meta-analysis also suggested that pubertal maturation was associated with only an increase of the intensity of emotional affect in the family conflicts but not the frequency of the conflicts^2^. Thus, we believe that puberty plays a critical role in modulating the HPA reactivity in response to the chronic family conflict. which is in parallel with the emotional amplification of family conflict across adolescent period^2^. Nonetheless, some inconsistent findings have been noted previously as adverse family environments (e.g. maternal smoking^39^, child conflict at home^14^) have been reported to affect the basal cortisol levels and cortisol reactivity even in early childhood^10^,^14^,^39^. Quevedo *et al.* reported that adverse care early in life was associated with a blunted cortisol awakening response in the pre/early pubertal adolescents, but not in the late pubertal adolescents^40^. Nonethesless, further studies are warranted to clarify the intriguing relationships among puberty, HPA axis, and stressors^33^.

Finally, it is possible that the lack of association of family conflict with morning cortisol level in the pre/early pubertal adolescents might be due to the limited statistical power in this group. For example, puberty normally causes a rise in cortisol secretion, which allows sufficient variation for one to be able to observe an effect of family conflict on cortisol^37^. Further study with a large sample size is warranted to replicate our findings.

There is a lack of studies determining the potential sex differences in HPA axis response to family conflict. However, a number of studies have shown that females have stronger HPA axis response to various challenges, including examination stress^22^, work-related stressors^23^, and carbon dioxide or noise^24^. However, our study did not find any sex differences in the diurnal cortisol level between participants with high family conflict and those with low family conflict. Taken together, our findings support the hypothesis that the sex differences in the cortisol response to stress might vary with the type and chronicity of stressors^24^. In terms of family conflict, it seems that males and females show similar cortisol responses.

### Clinical implications

Family conflict is a common source of stressor in daily life. Recent review suggested that there is a significant effect of family conflict on children’s adaptation, stress reactivity (autonomic activity and HPA axis) and development of psychopathology^41^. We showed that the association between family conflict and a clinical diagnosis of mood and anxiety disorders only started to emerge in adults but not in adolescents, which implies that relevant interventions to ameliorate family conflict might be a preventive measure against the future development of mood and anxiety disorders in the young populations.

The merits of the study stemmed from several strengths. First, serial salivary samples, rather than a single sample, were collected to document the diurnal pattern of the cortisol levels. Second, some key potential confounding factors (e.g. sleep, mood, anxiety disorders, seasonality), which were not controlled for in most of the previous studies^42^, were taken into consideration in the analyses of the current study.

However, several limitations of the study should be noted. First, previous study has suggested a moderate day-to-day variability in salivary cortisol^43^. The measurement of salivary cortisol on one single day might limit the capability of reliably determining the diurnal cortisol levels, although we purposefully instructed the participants to collect the salivary samples during free days to minimize the effects of work- or study-related stress on the cortisol levels. Nonetheless, the diurnal salivary cortisol profile has been found to be rather stable in children and adolescents^35^. In addition, the cortisol wakening response (increased cortisol level within 30 minutes after awakening) was not obvious in our samples. This may raise the concerns about the accuracy of the timing of sample collection. Nonetheless, we have cross checked the wake up time by using actigraphy and/or sleep diary. Second, the findings should be considered in the context of the way we measured family conflict, which should be interpreted as chronic daily life conflict within a family. Our findings might not be generalized to other situations, such as acute stress. In addition, family conflict was reported by parents without further delineation of the dynamics and subtypes of family conflict (e.g. parent-parent, parent-offspring). The perception of family conflict might vary among individual family members. Third, the pubertal status was reported by the adolescents themselves rather than being rated by physician, albeit it has been suggested that self-reported Tanner stage has fairly good agreement with rater-rated assessment^28^. Fourth, participants from this study were recruited through a risk-stratification approach, with the original aims to explore the familial aggregation of insomnia^41^. In this regard, the rate of insomnia was relatively higher than that of the local general population due to this specific study design. The study design may limit the generalizability of the findings. Nonetheless, we have adjusted the effects of insomnia and other potential confounding factors in the statistical analyses. In addition, further analyses using the weighted data with consideration of the prevalence of insomnia in the general population did not show any discrepancy in terms of the major findings. Finally, about 10% of the serial salivary cortisol data were reported as missing, which could potentially lead to a bias in the findings. However, additional analyses without imputation showed that the findings are consistent with those based on the analyses with imputation.

In summary, adverse family environment is associated with HPA axis dysfunction in adults and late/post pubertal adolescents. Pubertal maturation may play a critical role in modulating the association of the level of family conflict with HPA axis function. Our findings have important implications with respect to the prevention and intervention of stress-related mental and physical health problems in those high-risk individuals who experience high family conflicts. However, the aforementioned limitations should be noted and the findings are preliminary. Further longitudinal community-based studies with larger sample size are warranted to replicate these findings.

## Additional Information

**How to cite this article**: Zhang, J. *et al.* Family conflict and lower morning cortisol in adolescents and adults: modulation of puberty. *Sci. Rep.* **6**, 22531; doi: 10.1038/srep22531 (2016).

## Supplementary Material

Supplementary Information

## Figures and Tables

**Table 1:** Comparisons in sample characteristics between participants with low and high family conflict.

	Adolescents (Probands and siblings)		P value
	Low family conflict (n = 153)	High family conflict (n = 52)	
Age, year (mean ± SD)	14.4 ± 3.2	13.9 ± 2.1	0.19
Female sex, %	54.2	45.3	0.26
BMI, kg/m^2^ (mean ± SD)	19.5 ± 3.1	19.5 ± 3.8	0.96
Waist-to-hip ratio, (mean ± SD)	0.79 ± 0.07	0.79 ± 0.07	0.69
Chronic use of medication, %	11.0	3.8	0.17^&^
Chronic medical conditions, %	16.8	26.4	0.12
Season of measure,%			
Spring	13.4	25.8	
Summer	28.3	9.1	0.001
Fall	39.0	31.8	
Winter	19.3	33.3	
Late/post pubertal status (Tanner stage 4 or 5), %	61.9	52.8	0.24
Insomnia diagnosis, %	25.8	32.1	0.38
Current mood disorder, %	2.6	0	0.57^&^
Current anxiety disorder, %	5.9	5.7	1.00^&^
Rising up time (mean ± SD)	8:18 ± 1:27	8:22 ± 0:59	0.81
	Adults (parents)		
	Low family conflict (n = 183)	High family conflict (n = 61)	
Age, year (mean ± SD)	46.3 ± 4.3	46.3 ± 3.7	0.96
Female sex, %	53.0	54.1	0.88
BMI, kg/m2 (mean ± SD)	24.1 ± 3.3	24.4 ± 3.3	0.54
Waist-to-hip ratio (mean ± SD)	0.86 ± 0.06	0.85 ± 0.07	0.35
Chronic use of medication, %	29.0	23.0	0.36
Chronic medical conditions, %	37.2	39.3	0.76
Season of measure,%			
Spring	15.0	29.4	
Summer	31.3	7.8	0.002
Fall	34.7	31.4	
Winter	19.0	31.4	
Insomnia diagnosis, %	29.0	26.2	0.68
Current mood disorders, %	3.8	13.1	0.014^&^
Current anxiety disorders, %	2.2	9.8	0.018^&^
Rising up time (mean ± SD)	7:33 ± 1:14	7:25 ± 1:03	0.47

**Table 2:** Differences in diurnal salivary cortisol levels between adult participants (parents) with low and high family conflict.

	Low family conflict, n = 183	High family conflict, n = 61	Mean difference ± SE	P value&
Awakening salivary Cortisol 0 min (nmol/l)	11.7	11.3	−0.5 ± 0.8	0.29
Awakening salivary Cortisol 30 min (nmol/l)	12.4	10.9	−1.6 ± 0.9	0.09
Awakening salivary Cortisol 60 min (nmol/l)	9.0	7.5	−1.5 ± 0.6	0.02
Awakening salivary Cortisol 90 min (nmol/l)	6.4	5.6	−0.8 ± 0.5	0.14
Salivary Cortisol at noon (nmol/l)	4.6	4.8	−0.1 ± 0.4	0.78
Salivary Cortisol at 4 pm (nmol/l)	3.9	3.7	0.3 ± 0.4	0.45
Salivary Cortisol at 10 pm (nmol/l)	2.3	2.1	−0.2 ± 0.2	0.31
AUC_g_ 30 min (nmol/l)	12.1	11.1	−1.0 ± 0.7	0.09
AUC_g_ 60 min (nmol/l)	22.8	20.2	−2.5 ± 1.3	0.04
AUC_g_ 90 min (nmol/l)	30.5	26.8	−3.7 ± 1.8	0.03
AUC_i_ 30 min (nmol/l)	0.3	−0.2	−0.5 ± 0.5	0.41
AUC_i_ 60 min (nmol/l)	−0.7	−2.2	−1.5 ± 1.2	0.36
AUC_i_ 90 min (nmol/l)	−4.8	−6.9	−2.2 ± 1.9	0.43

^&^Adjustment for age, sex, chronic medication use, chronic medical condition, insomnia, current anxiety disorders, current depressive disorders, season, and wakeup time; Analyzed by GEE model in SPSS.

**Table 3:** Estimated means in diurnal salivary cortisol levels between adolescents with low and high family conflict and its interaction with pubertal status.

	Pre/early pubertal status		Mean difference ± SE	Late/post pubertal status		Mean difference ± SE	P value for family conflict	P value for puberty	P value for interaction
	Low family conflict, n = 58	High family conflict, n = 24		Low family conflict, n = 95	High family conflict, n = 28				
Awakening salivary Cortisol 0 min (nmol/l)	9.5	9.8	0.3 ± 1.0	11.2	9.6	−1.6 ± 1.2	0.41	0.10	0.18
Awakening salivary Cortisol 30 min(nmol/l)	10.0	10.5	0.5 ± 1.4	12.3	10.1	−2.2 ± 1.3	0.30	0.06	0.05
Awakening salivary Cortisol 60 min (nmol/l)	6.3	7.9	1.6 ± 1.0	8.4	7.9	−0.6 ± 0.9	0.46	0.01	0.01
Awakening salivary Cortisol 90 min (nmol/l)	4.4	5.9	1.5 ± 0.8	6.2	5.7	−0.5 ± 0.8	0.43	0.01	0.03
Salivary Cortisol at noon (nmol/l)	3.6	3.7	0.1 ± 0.5	4.4	3.8	−0.7 ± 0.5	0.25	0.09	0.16
Salivary Cortisol at 4 pm (nmol/l)	3.0	2.7	−0.3 ± 0.4	3.3	3.2	0.1 ± 0.4	0.44	0.14	0.18
Salivary Cortisol at 10 pm (nmol/l)	1.8	1.8	0 ± 0.3	1.9	1.9	0.1 ± 0.2	0.87	0.53	0.88
AUC_g_ 30 min (nmol/l)	9.9	9.9	0 ± 1.0	12.0	9.5	−3.9 ± 2.5	0.25	0.03	0.02
AUC_g_ 60 min (nmol/l)	18.0	18.9	0.9 ± 1.5	22.7	18.2	−4.5 ± 3.0	0.44	0.02	0.01
AUC_g_ 90 min (nmol/l)	23.3	26.3	3.0 ± 2.7	29.5	25.6	−3.9 ± 2.5	0.74	0.007	0.006
AUC_i_ 30 min (nmol/l)	0.0	0.3	0.3 ± 2.3	0.7	0.2	−0.5 ± 2.3	0.78	0.71	0.92
AUC_i_ 60 min (nmol/l)	−1.8	−0.2	1.6 ± 2.7	0.2	−0.5	−0.7 ± 2.6	0.83	0.68	0.94
AUC_i_ 90 min (nmol/l)	−5.2	−3.1	1.1 ± 3.1	−4.2	−3.3	0.9 ± 3.1	0.51	0.72	0.87

^$^Adjustment for sex, chronic medication use, chronic medical condition, insomnia, current anxiety disorders, current depressive disorders, seasonality and wakeup time; Analyzed by GEE model in SPSS.
