# Supplementary material for: Family conflict and lower morning cortisol in adolescents and adults: modulation of puberty
Source: Sci Rep. 2016 Mar 1;6:22531. doi: 10.1038/srep22531 (PMC4772378; doi:10.1038/srep22531)
Supplement: Supplementary Information [file srep22531-s1.pdf]

Family conflict and lower morning cortisol in adolescents and adults: modulation of puberty

Short title: Family conflict and morning cortisol

Jihui Zhang[1,2], Siu-Ping Lam[1], Alice PS Kong[3], Ronald CW Ma[3], Shirley Xin Li[4], Joey WY Chan[1], Mandy WM Yu[1], Junying Zhou[1], Michael HM Chan[5], Chung-Shun Ho[5], Albert M Li[6], Xiangdong Tang[7], Yun-Kwok Wing[1,2]*

[1]Department of Psychiatry, Faculty of Medicine, The Chinese University of Hong Kong, Hong Kong SAR, China;

[2]Shenzhen Research Institute, The Chinese University of Hong Kong;

[3]Department of Medicine and Therapeutics, Faculty of Medicine, The Chinese University of Hong Kong, Hong Kong SAR, China;

[4]Department of Psychology, The University of Hong Kong, Pokfulam Road, Hong Kong SAR, China

[5]Department of Chemical Pathology, Faculty of Medicine, The Chinese University of Hong Kong, Hong Kong SAR, China;

[6]Department of Paediatrics, Faculty of Medicine, The Chinese University of Hong Kong, Hong Kong SAR, China;

[7]Sleep Medicine Center, Mental Health Center, West China Hospital, Sichuan University, Chengdu, China.

Supplementary table 1: Associations of diurnal cortisol levels with family conflict in adults

	Coefficient	Standard error	Z value	P value
Awakening salivary Cortisol 0 min	-1.28	.78	-1.65	0.10
Awakening salivary Cortisol 30 min	-2.70	.97	-2.79	0.005*
Awakening salivary Cortisol 60 min	-1.58	.67	-2.35	0.019*
Awakening salivary Cortisol 90 min	-1.00	.56	-1.78	0.075
Salivary Cortisol at noon	.06	.46	0.14	0.89
Salivary Cortisol at 4 pm	-.29	.56	-0.52	0.61
Salivary Cortisol at 10 pm	-.52	.29	-1.80	0.07
AUC_g 30 min (nmol/l)	-1.89	.64	-2.94	0.003*
AUC_g 60 min (nmol/l)	-3.90	1.32	-2.96	0.003*
AUC_g 90 min (nmol/l)	-5.24	1.81	-2.89	0.004*
AUC_i 30 min (nmol/l)	-.40	.51	-0.79	0.43
AUC_i 60 min (nmol/l)	-.93	1.39	-0.67	0.50
AUC_i 90 min (nmol/l)	-.86	2.07	-0.42	0.68

Adjustment for age, sex, chronic medication use, chronic medical condition, insomnia, current anxiety disorders, current depressive disorders, season, and wakeup time; weighting for insomnia prevalence in general adults population (10%); * $p < 0.05$

Supplementary table 2: Associations of diurnal cortisol levels with family conflict in adolescents at late/post pubertal status

	Coefficient	Standard error	Z value	P value
Awakening salivary Cortisol 0 min	-2.00	1.12	-1.77	0.08
Awakening salivary Cortisol 30 min	-4.29	1.49	-2.88	0.004*
Awakening salivary Cortisol 60 min	-1.83	1.15	-1.59	0.11
Awakening salivary Cortisol 90 min	-.65	.88	-0.74	0.46
Salivary Cortisol at noon	-1.33	.52	-2.55	0.011*
Salivary Cortisol at 4 pm	-.37	.53	-0.69	0.49
Salivary Cortisol at 10 pm	-.37	.37	-1.00	0.32
AUC_g 30 min (nmol/l)	-2.30	1.15	-2.00	0.046*
AUC_g 60 min (nmol/l)	-4.11	2.10	-1.95	0.051
AUC_g 90 min (nmol/l)	-5.56	2.67	-2.08	0.037*
AUC_i 30 min (nmol/l)	-.52	.52	-0.99	0.32
AUC_i 60 min (nmol/l)	-.73	1.49	-0.49	0.62
AUC_i 90 min (nmol/l)	.35	2.39	0.15	0.88

Adjustment for age, sex, chronic medication use, chronic medical condition, insomnia, current anxiety disorders, current depressive disorders, season, and wakeup time; Weighting for insomnia prevalence in general adolescent population (6%); * $p < 0.05$

Supplementary table 3: Associations of diurnal cortisol levels with family conflict in adolescents at pre/early pubertal status

	Coefficient	Standard error	Z value	P value
Awakening salivary Cortisol 0 min	-.38	1.36	-0.28	0.78
Awakening salivary Cortisol 30 min	1.36	2.24	0.61	0.54
Awakening salivary Cortisol 60 min	1.31	1.44	0.91	0.36
Awakening salivary Cortisol 90 min	1.50	1.04	1.45	0.15
Salivary Cortisol at noon	.84	1.08	0.79	0.43
Salivary Cortisol at 4 pm	1.30	.98	1.32	0.18
Salivary Cortisol at 10 pm	-.03	.24	-0.12	0.91
AUC_g 30 min (nmol/l)	.09	.24	0.38	0.70
AUC_g 60 min (nmol/l)	.39	.49	0.81	0.42
AUC_g 90 min (nmol/l)	.74	.62	1.19	0.23
AUC_i 30 min (nmol/l)	.10	.14	0.70	0.48
AUC_i 60 min (nmol/l)	.40	.41	0.98	0.33
AUC_i 90 min (nmol/l)	.75	.63	1.19	0.24

Adjustment for age, sex, chronic medication use, chronic medical condition, insomnia, current anxiety disorders, current depressive disorders, season, and wakeup time; Weighting for insomnia prevalence in general adolescent population (6%); * $p < 0.05$
